# One-Step Ball Milling Preparation of Nanoscale CL-20/Graphene Oxide for Significantly Reduced Particle Size and Sensitivity

**DOI:** 10.1186/s11671-017-2416-y

**Published:** 2018-02-07

**Authors:** Baoyun Ye, Chongwei An, Yuruo Zhang, Changkun Song, Xiaoheng Geng, Jingyu Wang

**Affiliations:** 1 School of Environment and Safety Engineering, North University of China, Taiyuan, 030051 China; 2 Shanxi Engineering Technology Research Center for Ultrafine Powder, North University of China, Taiyuan, 030051 China; 3 The 213th Research Institute of China Ordnance Industry, Xi’an, 710061 China; 4 Department of Chemical Engineering and Safety, Binzhou University, Binzhou, 256603 China

**Keywords:** CL-20, Graphene materials, Ball milling, Impact sensitivity

## Abstract

A one-step method which involves exfoliating graphite materials (GIMs) off into graphene materials (GEMs) in aqueous suspension of CL-20 and forming CL-20/graphene materials (CL-20/GEMs) composites by using ball milling is presented. The conversion of mixtures to composite form was monitored by scanning electron microscopy (SEM) and powder X-ray diffraction (XRD). The impact sensitivities of CL-20/GEM composites were contrastively investigated. It turned out that the energetic nanoscale composites based on CL-20 and GEMs comprising few layers were accomplished. The loading capacity of graphene (reduced graphene oxide, rGO) is significantly less than that of graphene oxide (GO) in CL-20/GEM composites. The formation mechanism was proposed. Via this approach, energetic nanoscale composites based on CL-20 and GO comprised few layers were accomplished. The resulted CL-20/GEM composites displayed spherical structure with nanoscale, ε-form, equal thermal stabilities, and lower sensitivities.

**Electronic supplementary material:**

The online version of this article (10.1186/s11671-017-2416-y) contains supplementary material, which is available to authorized users.

## Background

Unintentional detonation of munitions from accidents and asymmetric threats must be presented to eliminate the loss of innocent lives and infrastructure in modern conflicts [1]. Thereinto, high explosives (HEs) including 2,4,6,8,10,12-hexanitro-2,4,6,8,10,12-hexaazaisowurtzitane (HNIW or CL-20), 1,3,5,7-teranitro-1,3,5,7-tetrazocine (HMX), and hexahydro-1,3,5-trinitro-1,3,5-triazine (RDX) exhibiting high energy usually encounter poor sensitivity towards impact, friction, shock wave, thermo, and electric spark [2]. During the past decades, considerable works have been carried out to design and synthesize insensitive energetic simple substance or composites [3–6]. There are four main route for the synthesis of energetic composites: preparing the polymer-bonded explosives (PBXs) via coating (including aqueous suspension and spray drying) [7–9], fabrication of microcapsules via in situ polymerization [2], and exploring energetic cocrystals [10]. The technique of coating is the most common method of energetic composite synthesis. However, this approach is not environmentally friendly owing to the use of large amounts of solvent. Explosives based on NC [11], estane [12], EPDM [13], etc. have been reported using this route. In situ polymerization was the method reported by Yang [2] which led to the three typical nitramine explosive-based microcapsules exhibiting obvious core-shell structures. The explosive is swollen in the reaction process of polymerization to generate composites. However, the shielding gas is required due to the danger of the explosive. Furthermore, the particle size of the composite is therefore difficult to control. Co-crystals have demonstrated great potential in various important applications by adjusting crystal structure at the molecular level in the material science. Qiu et al. reported a way of producing the nanoscale cocrystal of 2CL-20·HMX by bead milling an aqueous suspension of CL-20 and HMX in a stoichiometric ratio of the cocrystal. The reported method is deemed to be a potential in advancing the production and application of energetic cocrystalline materials [14]. However, the newly developed energetic materials are still not able to completely replace the currently used HEs due to various problems including chemical incompatibility, instability, and high sensitivity [15].

Inspired by the advantages of the method, the nanoscale CL-20-based composite was first produced in this paper. The particle size, size distribution, and morphology of the explosives are essential physical characteristics which significantly influence their sensitivities. Explosives with a small particle size, narrow size distribution, and rounded morphology exhibit markedly lowered initiation sensitivity and reduced critical diameter. However, it is quite being difficult to produce nanoscale particles from traditional method including solvent-nonsovent recrystallization, sol-gel, and spray drying and supercritical fluid technique [16]. The abovementioned methods are effective at the lab scale, and the large-scale preparation of nanoscale energetic materials involves great difficulties. Mechanical ball milling (also known as ball milling) is a desirable choice because it is suitable for massive and continuous preparation of uniform morphology crystals that maintained the original crystal form.

Graphene, since its emergence in 2004, possessing many desired properties including superior thermal and electrical conductivities, good lubrication, and excellent mechanical properties has been intensively studied. Graphene oxide (GO) is an intermediate in the chemical route to graphene (reduced graphene oxide, rGO). As a hydrophilic two-dimensional monolayer material, GO has been extensively used in emulsifiers, membranes, and sorbents. For a long time, GO has been considered as an energetic material with thermal instability [17]. It has been reported that graphene materials (GEMs) including graphene oxide and graphene could stabilize explosives such as HMX, RDX, CL-20, and styphnate [8, 18–21]. Previously, we used graphene oxide to reduce the impact and shock wave sensitivities, obtaining an excellent insensitive HMX/Viton/GO composite for booster explosive via water-suspension method [8]. Compared with this method above, mechanical ball milling possesses large-scale manufacturing and commercialization of bulk composites which is an ideal method for achieving superior morphology and small particle of products. Furthermore, dried graphene oxide is easy to aggregate graphite oxide. When the number of graphene layer is greater than 10, the electron energy band structure of graphene is approaching its three-dimensional limit. It is very important controlling GO or rGO with a selected number of layers to keep the special properties of two-dimensional materials.

In this work, we report a novel method of mechanical ball milling to prepare nanoscale CL-20-based composite using GEMs. This method can exfoliate graphite materials into graphene materials, save the trouble of exfoliation in preparing graphene materials, and minimize aggregate-induced nanosheet-nanosheet interactions.

## Methods

### Synthesis of Nanoscale CL-20/GEMs Composites

The aqueous suspension was prepared by adding raw CL-20 (purchased from Liaoning Qingyang Chemical Industry Co., Ltd.) or mixtures of raw CL-20 and additives (graphite materials (GIMs)) in various weight ratios were milled, with the aim to synthesize nanoscale CL-20 or CL-20/GEMs composites, respectively. The schematic of the bead milling process is shown in Additional file 1: Figure S1. The milling conditions were as follows: sample mass—10 g (the ratios of raw CL-20 and additives were 99.5:0.5, 99:1, 98:2, and 95:5, and the samples with different weight percentages were denoted as milling CL-20, CL-20/GO_0.5_, CL-20/GO_1_, CL-20/GO_2_, CL-20/GO_5_, CL-20/rGO_1_, and CL-20/rGO_5_), zirconia balls with the diameter 0.1 mm, ball-to-powder ratio 20, rotation speed of the planet carrier—300 RPM, medium—de-ionized, and de-ionized to powder ratio 10. The ground powder was used for sonication to remove completely the zirconia balls from the product. See in Additional file 1: Experimental Details for further details on the methods for the synthesis of the graphite oxide and graphene.

### Characterization

Field-emission scanning electron microscopy (FESEM) images were taken on a MIRA3 LMH SEM (Tescan) at 10 k. X-ray diffraction (XRD) patterns were obtained using a DX-2700 (Dandong Haoyuan Corporation, Liaoning, China) X-ray diffractometer with Cu-Kα (40 kV, 30 mA) radiation at λ = 1.5418 Å. All samples were scanned from 5° to 50° with steps 0.03 and 6 s counting time. Thermal analysis was performed on a differential scanning calorimeter (DSX-131, France Setaram Corporation, Shanghai, China) at heating rates of 5, 10, and 20 °C/min. The impact sensitivity was tested with a home-built type 12 drop hammer apparatus. The special height (H_50_) represents the height from which 2.500 ± 0.002 kg drop-hammer will result in an explosive event in 50% of the trials. In each determination, 25 drop tests were made to calculate the H_50_.

## Results and Discussion

**Fig. 1:**
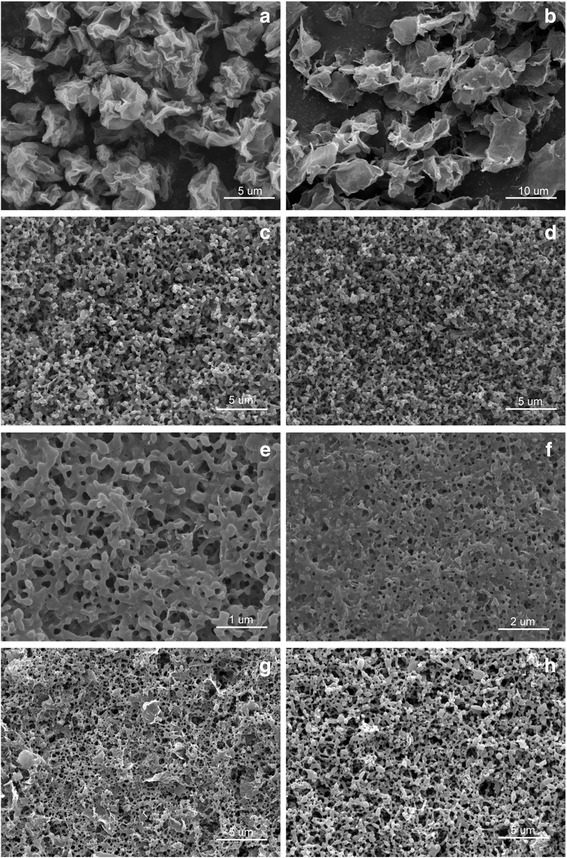
SEM images of samples. **a** GO. **b** rGO. **c** milling CL-20. **d** CL-20/GO_0.5_. **e** CL-20/GO_1_. **f** CL-20/GO_2_. **g** CL-20/GO_5_. **h** CL-20/rGO_5_

The morphologies of the as-synthesized samples were studied by SEM, and the results are shown in Fig. 1 and Additional file 1: Figure S2. Additional file 1: Figure S2a shows that graphite oxide appears typical layer structure similar to flake graphite (Additional file 1: Figure S2b). It is different from that of graphene oxide (Fig. 1a) which displays flaky morphology with some wrinkles and folding on the surface and edge. Scrolling and corrugation are part of the intrinsic nature of GO sheets, which result from the fact that the 2D membrane structure becomes thermodynamically stable via bending [22]. Figure 1b shows that the graphene sheets are highly transparent with folding at the edges, suggesting a very small thickness. Because of the high specific area, the graphene sheets aggregated and formed a stacked graphitic structure when they were dried.

SEM images of CL-20/GEMs composites are shown in Fig. 1c–f, and the SEM image of raw CL-20 is shown in Additional file 1: Figure S1c. It can be seen that most of the milled CL-20 microparticles form sphere shape with smooth surface after ball milling, whereas the starting material presents spindle shape (Additional file 1: Figure S2c). Additionally, the average particle size of the milled CL-20 is 200 nm, which is clearly smaller than that of the raw CL-20 (300 μm). The differences in their morphology from Fig. 1c to Fig. 1e are obvious. After addition of graphite oxide, some wrinkles are observed on its surface. This reveals that GO sheets are deposited on the surface of CL-20 during the ball milling process. It follows from SEM results presented that the retention rate of GO increased with increasing the addition of graphite oxide. However, graphene sheets in CL-20/rGO_5_ are not being detected clearly in Fig. 1f. The main reason for this result is discussed in following part.

**Fig. 2:**
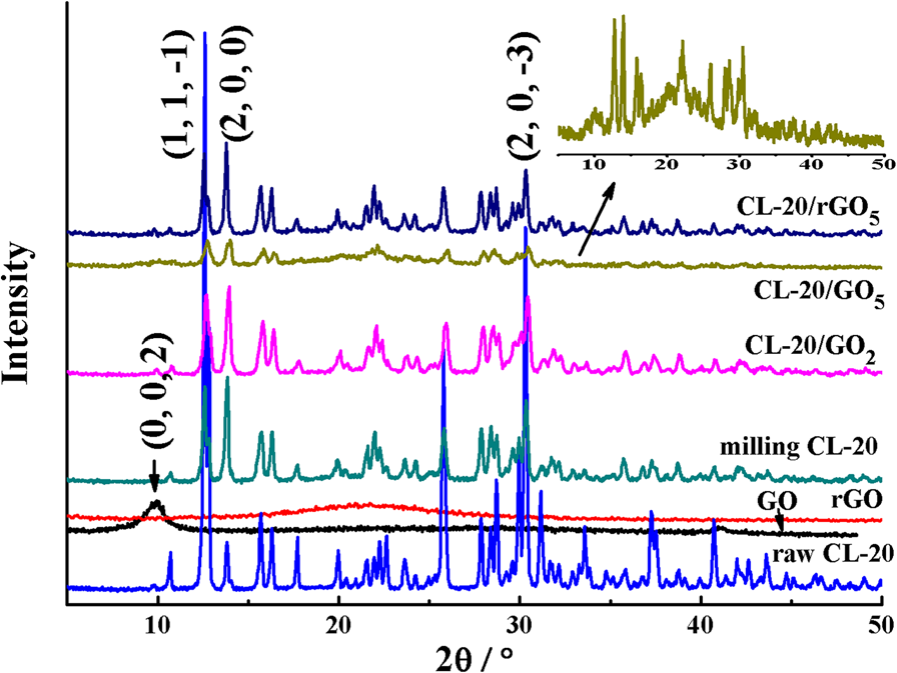
XRD spectra of GO, rGO, CL-20, and CL-20/GEMs

XRD analyses were carried out to investigate the crystal structure of as-prepared samples. The XRD curves of raw CL-20, CL-20/GEMs, GO, and rGO are shown in Fig. 2, and the magnified curve of CL-20/GO_5_ is displayed in the inset (Additional file 1: Figure S3 displays the XRD curves of flake graphite and graphene). Raw CL-20 displays three characteristic diffraction peaks at 12.59°, 13.82°, and 30.29°, attributed to the crystal plane (1, 1, − 1), (2, 0, 0), and (2, 0, − 3), respectively (PDF Card 00-050-2045). The results suggest that the diffraction peaks of milling specimens are corresponded well with that of raw CL-20. It can be also observed that the diffraction intensity of milling CL-20 and CL-20/GEMs is visibly decreased after milling, while the intensity of 13.81° (2, 0, 0) is relatively increased. This is probably because of the preferred orientation caused by the effect of ball milling. For CL-20/GO_5_, the typical diffraction peak of GO at 10° (0, 0, 2) is observed, showing the presence of GO. However, in the XRD curve of CL-20/GO_2_, there are no noticeable diffraction peaks detected because of lower GO content. Moreover, compared with CL-20, the peaks in CL-20/rGO_5_ have no obvious difference. The result is consistent with that of SEM.

**Fig. 3:**
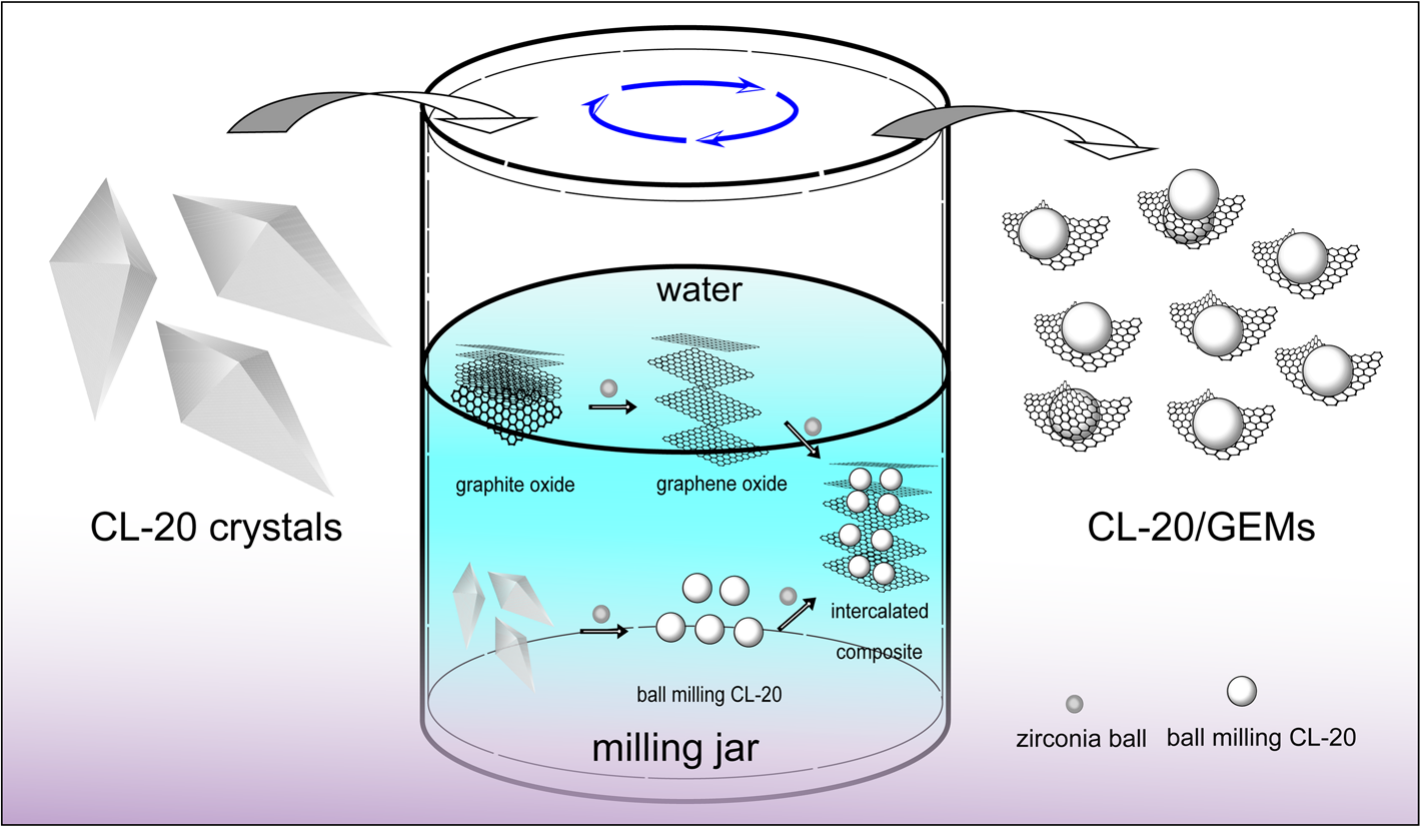
Schematic of formation of CL-20/GO composite

The formation mechanism during ball milling was proposed, and the schematic was illustrated in Fig. 3. The main reason for this phenomenon is proposed below. The formation of CL-20/GEMs could be divided into two processes: exfoliation of GIMs and refining of CL-20, respectively, and formation of intercalated composites. It is easy to form non-covalent bond structures between CL-20 and GO because of the functional group (–OH, –COOH, and –C–O–C) existed in GO. However, the situation is different for rGO because of little functional group in rGO. Detail formation mechanism is summarized in Additional file 1.

**Fig. 4:**
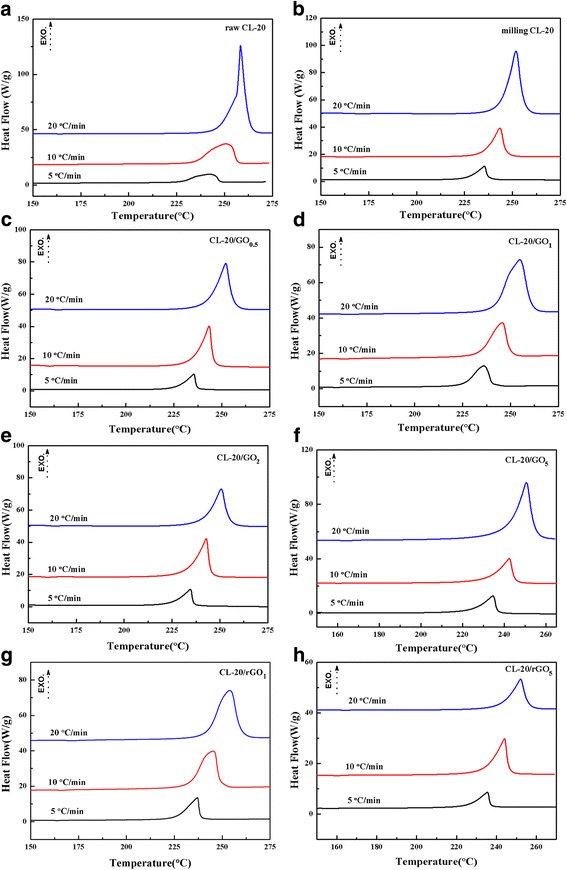
**a**–**h** DSC curves of as-prepared samples collected at different heating rates. **a** raw CL-20, **b** milling CL-20, **c** CL-20/GO_0.5_, **d** CL-20/GO_1_, **e** CL-20/GO_2_, **f** CL-20/GO_5_, **g** CL-20/rGO_1_, **h** CL-20/rGO_5_

**Fig. 5:**
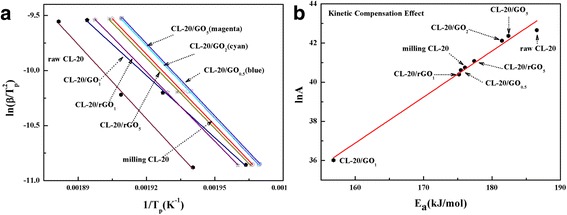
**a** Kissinger plots of ln(*β*/*T*_p_^2^) to 1/*T*_p_. **b** Kinetic compensation effect for thermal decomposition of as-prepared samples

The kinetic and thermodynamic parameters were very important in mastering the thermal properties of explosives. To investigate thermal performance of the nanocomposites, DSC traces collected at different heating rates were obtained in Fig. 4 and were used to calculate the parameters in Fig. 5 and Table 1. In the eight DSC traces at heating rates of 5, 10, and 20 °C/min, they have same trend in each curve. The decomposition heat increased as the heating rate increased, which is consistent with the usual case, i.e., HMX or RDX. From Fig. 4, it is easily found that the smooth decomposition curves in Fig. 4a changed to a truncated, asymmetric curve when raw CL-20 was heated at 20 °C/min (see top curve in Fig. 4a). This behavior represents the highly exothermic and accompanied by self-heating, which occurs when CL-20 decomposition reaction rate exceeds mass and heat transfer rates. This behavior is known to represent the explosive mode thermal decomposition. Thus, special safety issues arise during processing and storage routes based on CL-20. The DSC curves of nanoscale CL-20 were obtained smooth non-truncated heat curves, which indicate that nanocrystallization can reduce thermal runaway.

**Table 1:** Kinetics, thermal stabilities, and thermodynamics derived from DSC curves

Samples	Kinetics			Thermal stabilities		Thermodynamics		
	*E*_a_ (KJ/mol)	ln*A*	*R* ^2^	*T*_p0_/K	*T*_b_/K	ΔG^≠^(KJ/mol)	ΔH^≠^(KJ/mol)	ΔS^≠^(J/mol)
Raw CL-20	186.55	42.66	0.99	503.67	515.51	133.46	182.37	97.09
Milling CL-20	176.06	40.75	0.99	497.83	510.11	131.43	171.92	81.33
CL-20/GO_0.5_	175.46	40.61	0.99	498.05	510.39	131.41	171.32	80.13
CL-20/GO_1_	156.82	36.01	0.99	496.29	510.08	131.86	152.69	41.98
CL-20/GO_2_	181.45	42.12	0.99	496.50	508.34	131.27	177.32	92.74
CL-20/GO_5_	182.41	42.36	0.99	497.4	509.22	131.18	178.27	94.68
CL-20/rGO_1_	175.17	40.40	0.99	499.49	511.93	131.88	171.01	78.35
CL-20/rGO_5_	177.40	41.08	0.99	497.26	509.42	131.56	173.37	84.07

The Kissinger equation (Additional file 1: Eq. (1)) [8, 23] was enlisted to calculate the *E*_a_ (apparent activation energy) and *A* (pre-exponential factor) of samples. By contrasting the data of *E*_a_ and ln*A* in Table 1, the CL-20/GO_2_ and CL-20/GO_5_ composites show slightly higher *E*_a_ than others except raw CL-20. Figure 5a shows that the plots of milling CL-20 and CL-20/GEMs were close to each other, which may mean that they undergo similar decomposition reaction. The two dynamic parameters of the activation energy and the pre-exponential factor have the linear relationship of mutual compensation for the rate constant under certain conditions. The linear relationship between *E*_a_ and ln*A* can be explained with the Arrhenius equation (Additional file 1: Eq. (2)). Figure 5b shows the plot of ln*A* to *E*_a_, that is the kinetic compensation effect. The result implies that the milling CL-20 and CL-20/GEMs present good linear relationships (*R*^2^ > 0.99). This implies that the decomposition reactions of those samples have similar kinetic mechanisms in addition to raw CL-20.

The decomposition of CL-20/GEM composites complies with the decomposition mechanism of typical composite energetics consisting of solid fuels and oxidizers, such as pyrotechnics and composite propellants. In the CL-20/GO or CL-20/rGO nanocomposites, the oxidizer elements and fuel elements were incorporated into one molecule. Thus, the decomposition originates from the activation and rupture of its weakest bond. Those activation and rupture courses are very important to the thermal decomposition. Those courses dominate the entire decomposition process and can be described by the parameters of ΔG^≠^ (free energy of activation), ΔH^≠^ (enthalpy activation), and ΔS^≠^ (entropy of activation), which are calculated by Additional file 1: Eqs.(5)-(7) [24]. The meaning of ΔG^≠^ is the chemical potential of the activation course. Its values were positive numbers, which means that none of the activation courses proceeded spontaneously [25]. Therefore, those explosives are in a stable state in common condition. ΔH^≠^ is the absorb energy of the molecule from a stable state to the activated state. So the value of ΔH^≠^ was much closer to that of E_a_ for those samples. Comparing the data in Table 1, it was found that raw CL-20 needed the highest energy to be activated. However, in those nanoscale explosives, CL-20/GO_2_ and CL-20/GO_5_ have the highest energy which indicates that they need the highest energy to be activated. To investigate the thermal stability of raw CL-20 and nanoscale CL-20, the *T*_p0_ (peak temperature when *β*_i_ is zero) and *T*_b_ (critical explosion temperature) were obtained by Additional file 1: Eqs. (3) and (4) [26, 27]. From Table 1, the nanoscale CL-20 presented equivalent thermal stability, which implies that GO or rGO have little influence on the thermal stability of CL-20.

To forecast the safety performance of samples, the test of impact sensitivities was performed, and the results are presented in Fig. 5. It should be noted that the special height (H_50_) of as-prepared samples is higher than that of raw CL-20, probably because the grain size of explosives influences the impact sensitivity significantly. As to raw CL-20 and milling CL-20, they can be concluded that excellent desensitization effect has been achieved for the improved crystal morphologies and grain-size distribution by ball milling method, especially compared with refining CL-20 prepared by solvent-nonsolvent method [28].

**Fig. 6:**
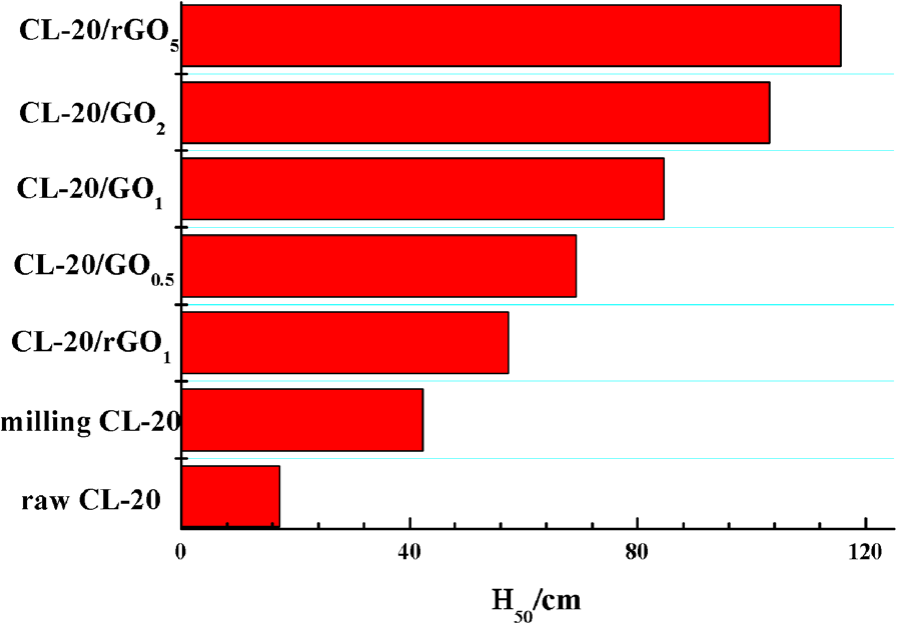
Impact sensitivities of CL-20 before and after milling. The impact sensitivities of raw CL-20, milling CL-20 and CL-20/GEMs with various content of GEMs are shown in Additional file 1: Table S1

The impact sensitivities of CL-20 with different content of GEMs are lower than that of milling CL-20 (Fig. 6). The reduced impact sensitivities of CL-20/GEMs are supposed from the excellent lubrication and heat conduction of GEMs, which could reduce the internal folding dislocations and hot spots [9, 19]. Moreover, the impact sensitivities reduced with the increase of GEM content. However, the impact sensitivity of CL-20/GO_1_ differs from CL-20/rGO_1_ despite with the same content of GEMs. The special height of CL-20/rGO_5_ reaches 120 cm, while the H_50_ of CL-20/GO_5_ exceeds 150 cm. The different load capacity is the main reason for this phenomenon, and these results verify the hypothesis proposed above, and the specific data value is shown in Additional file 1: Table S1.

## Conclusions

In conclusion, we put forward a scalable ball milling technique to produce CL-20/GEM composites with nanoscale grain size, equal thermal stabilities, and reduced impact sensitivities. The formation mechanism between CL-20 and GEMs is proposed. The oxygen functional groups in GO facilitate the production of CL-20/GO due to formation of hydrogen bonding interactions with CL-20, consequently producing graphene oxide, and minimizing re-aggregation. In addition, this method is a very useful way for exfoliating graphene oxide from graphite oxide, avoiding tedious works in preparing graphene oxide. This method could be easily applied to other materials (e.g., graphene oxide load metal or polymer) to produce graphene oxide-based composites. The as-prepared CL-20/GEM composites are very suitable as the main ingredient in booster or propellant.

## Additional file

Additional file 1: Supporting Information for One-step ball milling preparation of nanoscale CL-20/graphene oxide for significantly reduced particle size and sensitive. (DOCX 1448 kb)

## Abbreviations

2D: Two-dimensional
CL-20: 2,4,6,8,10,12-hexanitro-2,4,6,8,10,12-hexaazaisowurtzitane
E_a_: Apparent activation energy
EPDM: Ethylene-propylene-diene monomer
FESEM: Field-emission scanning electron microscopy
GEMs: Graphene materials
GIMs: Graphite materials
GO: Graphene oxide
H_50_: Special height
HEs: High explosives
HMX: 1,3,5,7-teranitro-1,3,5,7-tetrazocine
NC: Nitrocellulose
PBXs: Polymer-bonded explosives
PDF: Portable document format
RDX: Hexahydro-1,3,5-trinitro-1,3,5-triazine
rGO: Reduced graphene oxide/graphene
*T*_b_: Critical explosion temperature
*T*_p0_: Peak temperature when *β*_i_ is zero
XRD: X-ray diffraction
ΔG^≠^: Free energy of activation
ΔH^≠^: Enthalpy of activation
ΔS^≠^: Entropy of activation
