# Supplementary material for: One-Step Ball Milling Preparation of Nanoscale CL-20/Graphene Oxide for Significantly Reduced Particle Size and Sensitivity
Source: Nanoscale Res Lett. 2018 Feb 7;13:42. doi: 10.1186/s11671-017-2416-y (PMC5803152; doi:10.1186/s11671-017-2416-y)
Supplement: Additional file 1: — Supporting Information for One-step ball milling preparation of nanoscale CL-20/graphene oxide for significantly reduced particle size and sensitive. (DOCX 1448 kb) [file 11671_2017_2416_MOESM1_ESM.docx]

**Supporting Information**

**One-step ball milling preparation of nanoscale CL-20/graphene oxide for significantly reduced particle size and sensitivity**

Baoyun Ye^a^, Chongwei An^a,b,^*, Yuruo Zhang^c^, Changkun Song^a^, Xiaoheng Geng^d^, Jingyu Wang^a,b^

^a^ School of Environment and Safety Engineering, North University of China, 030051 Taiyuan, China

^b^ Shanxi Engineering Technology Research Center for Ultrafine Powder, North University of China, 030051 Taiyuan, China

^c^ The 213th Research Institute of China Ordnance Industry, 710061 Xi’an, China

^d^ College of Resources and Environmental Engineering, Binzhou University, 256603 Binzhou, China

**^*^ Corresponding author**, Email address: [anchongwei@yeah.net](mailto:anchongwei@yeah.net) (Chong Wei An)

**Experimental Details**

**1. Preparation of graphene oxide**

Graphite oxide (GO) used in this work was prepared from flake graphite according to modified Hummers’ method [1]. Flake graphite (2 g) and NaNO_3_ (1.5 g) were placed in a 1000 mL breaker. Concentrated sulfuric acid (H_2_SO_4_, 150 mL) was then added with stirring in an ice-water bath, the KMnO_4_ (9 g) was slowly added over 30 min. After 2 h of stirring, the beaker was transferred to an oil bath at 40 °C for 5 days with vigorous electric stirring. At last, 30 wt. % H_2_O_2_ (6 mL) was added, and the mixture was stirred for 2 h at room temperature. The mixture was washed thoroughly with a mixed aqueous solution of HCl/acetone. After filtering, washing and drying, graphite oxide was obtained.

The graphite oxide obtained above was added into deionized water to form a typical graphite oxide suspension (1mg/mL). The graphite oxide suspension was used ultrasonic probe over 40min at room temperature to exfoliate graphene oxide (GO) from graphite oxide. The suspension was then centrifuged (3000 rpm for 10min), and the supernatant was dried by freeze dryer, known as graphene oxide.

**2. Preparation of graphene**

In a typical synthesis to prepare graphene (rGO) directly from graphene oxide, graphene oxide (0.5 g) was added into deionized water (200 mL) to form a homogeneous yellow-brown dispersion under ultrasonication condition. 80 wt.% Hydrazine hydrate (5 mL) was added and the solution heated in an oil bath at 100 °C for 2 h over which the reduced graphene oxide gradually precipitated out as a black solid. Precipitation was filtered, washed with deionized water, and dried in freezer dryer, known as graphene.

**Additional Results**


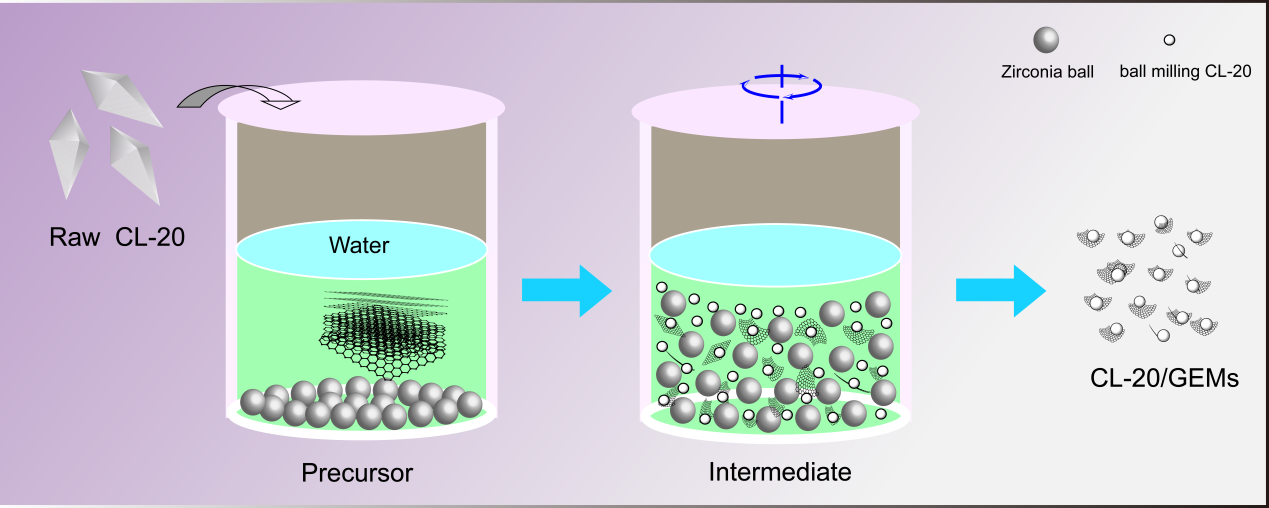


Figure S1. The schematic of the bead milling process to prepare CL-20/GEMs composites.


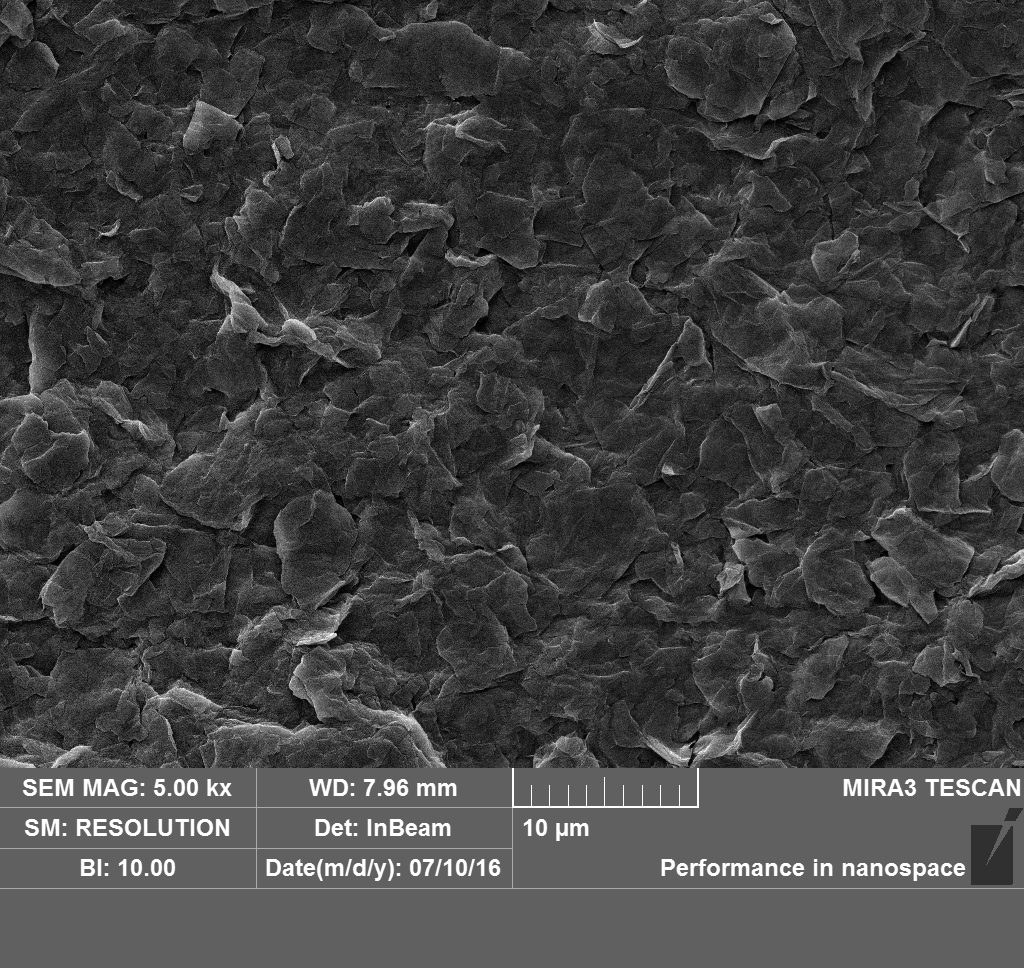

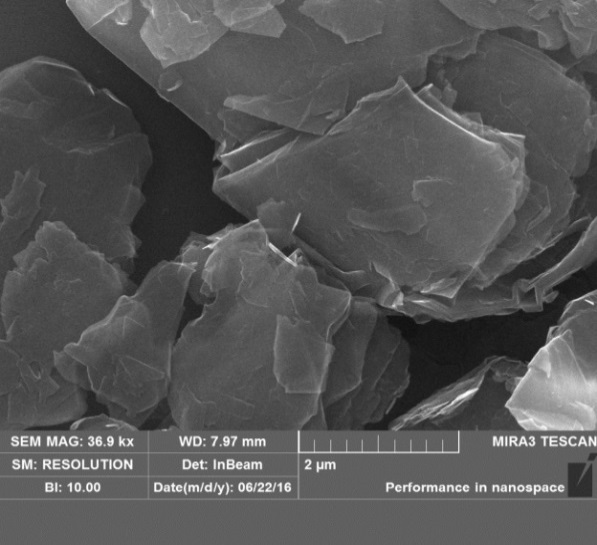


(a) (b)


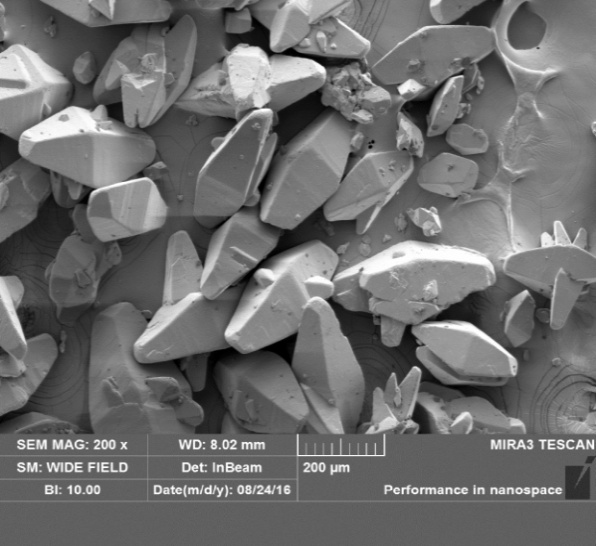


(c)

Figure S2. SEM images of (a) graphite oxide, (b) flake graphite, and (c) raw CL-20.


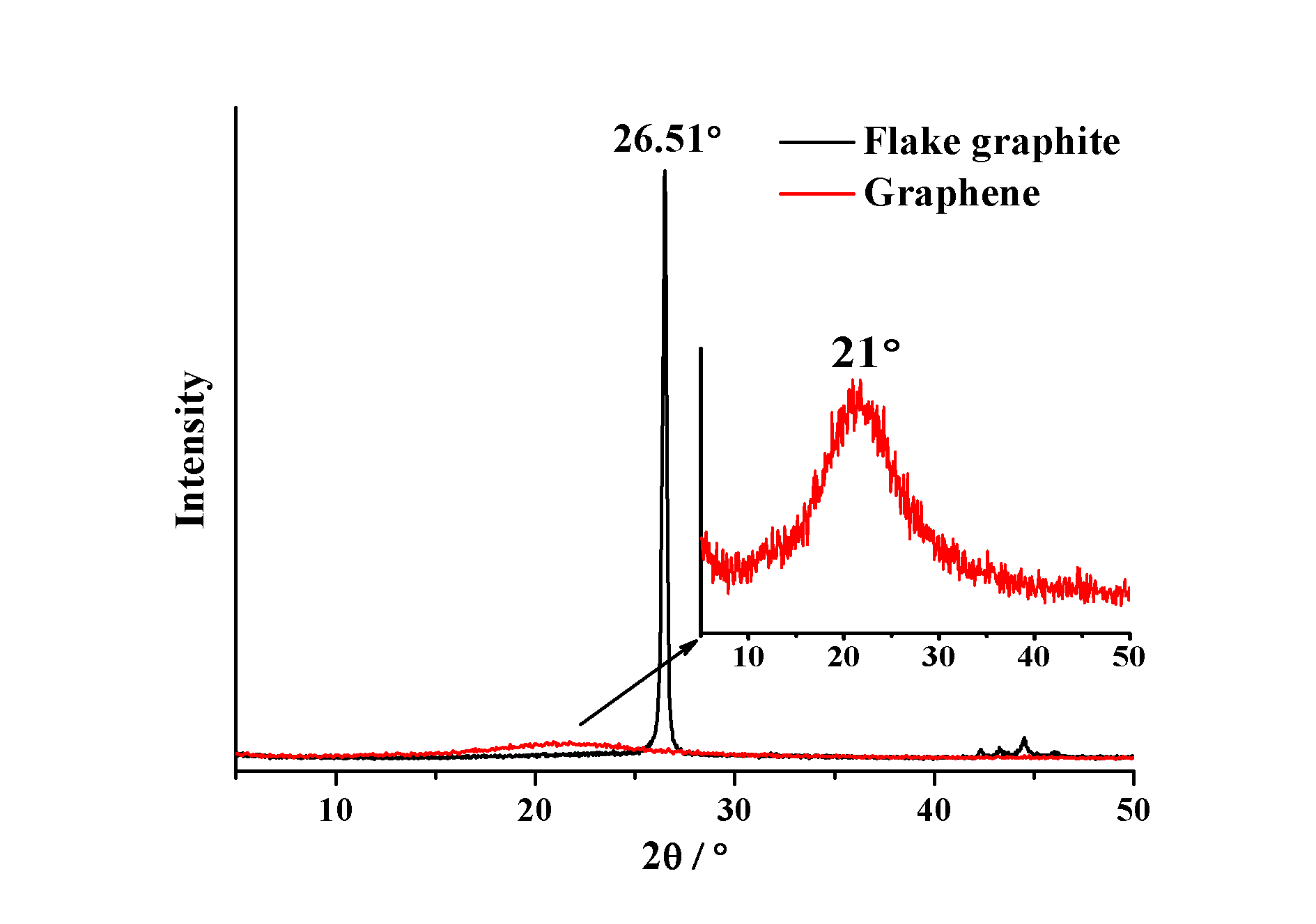


Figure S3. XRD curves of flake graphite and graphene.

Table S1. Impact sensitivities of raw CL-20, milling CL-20 and CL-20/GEMs with various content of GEMs.

| samples | Special Height (H_50_)/cm |
| --- | --- |
| Raw CL-20 | 17.3 |
| Milling CL-20 | 42.3 |
| CL-20/rGO_1_ | 57.4 |
| CL-20/GO_0.5_ | 69.2 |
| CL-20/GO_1_ | 84.6 |
| CL-20/GO_2_ | 103.2 |
| CL-20/rGO_5_ | 115.7 |
| CL-20/GO_5_ | ﹥150 |

**3.** **Mechanism**

The formation of CL-20/GEMs could be divided into two processes: exfoliation of GIMs and refining of CL-20 respectively and formation of intercalated composites. GIMs layers are arranged to form bulk GIMs via Van der Waals forces (VDW). These forces may resist the exfoliation of GEMs from GIMs via milling process. The impact and shear forces generated via high energy ball milling can overcome the forces between the GIMs layers [2]. These shear and impact forces can deliver an energy amount required to reduce the VDW between the GIMs layers and finally introduce facile exfoliation of the GEMs sheets [3]. In this work, the GEMs were exfoliated layer by layer from GIMs according to the above mechanism that was enhanced via application of water as a milling environment to provide a great support for non-destructive exfoliation [2]. Meanwhile, the impact and shear force produced by the ball milling would make for milling CL-20. The obtained CL-20 then inserted into GEMs layers to form the intercalated composite.

The oxygen functional group in GO including hydroxyl (-OH), carboxyl (-COOH), epoxy group (-C-O-C), etc. Among these groups, hydroxyl and carboxyl can form hydrogen bonds with nitro in CL-20 under the action of ball mill. Hydrogen bonds enable GO and CL-20 to form CL-20/GO composite material. RGO, in contrast to GO, is difficult to interact with CL-20 due to the little oxygen functional group. Therefore, there is only a small amount of rGO detected on the surface of CL-20/rGO5, although the high value of rGO.

**4. Equations**

$\boldsymbol{ln}\frac{\boldsymbol{\beta}_{\boldsymbol{i}}}{\boldsymbol{T}_{\boldsymbol{p}}^{\boldsymbol{2}}}\boldsymbol{=ln}\frac{\boldsymbol{R\times A}}{\boldsymbol{E}_{\boldsymbol{a}}}\boldsymbol{-}\frac{\boldsymbol{E}_{\boldsymbol{a}}}{\boldsymbol{R}}\boldsymbol{\times}\frac{\boldsymbol{1}}{\boldsymbol{T}_{\boldsymbol{p}}}$ (1)

$\mathbf{k=}\boldsymbol{A}_{\boldsymbol{k}}\boldsymbol{\times Exp}\left( \boldsymbol{-}\frac{\boldsymbol{E}_{\boldsymbol{k}}}{\boldsymbol{T}_{\boldsymbol{p}\boldsymbol{0}}\boldsymbol{\times R}} \right)$ (2)

$\boldsymbol{T}_{\boldsymbol{pi}}\boldsymbol{=}\boldsymbol{T}_{\boldsymbol{p}\boldsymbol{0}}\boldsymbol{+b}\boldsymbol{\beta}_{\boldsymbol{i}}\boldsymbol{+c}\boldsymbol{\beta}_{\boldsymbol{i}}^{\boldsymbol{2}}\boldsymbol{+d}\boldsymbol{\beta}_{\boldsymbol{i}}^{\boldsymbol{3}}$ (3)

$\boldsymbol{T}_{\boldsymbol{b}}\boldsymbol{=}\frac{\boldsymbol{E}_{\boldsymbol{a}}\boldsymbol{-}\sqrt{\boldsymbol{E}_{\boldsymbol{a}}^{\boldsymbol{2}}\boldsymbol{-4}\boldsymbol{R}\boldsymbol{E}_{\boldsymbol{a}}\boldsymbol{T}_{\boldsymbol{P}\boldsymbol{0}}}}{\boldsymbol{2}\boldsymbol{R}}$ (4)

$\mathbf{Aexp}\left( \boldsymbol{-}\frac{\boldsymbol{E}_{\boldsymbol{a}}}{\boldsymbol{RT}} \right)\boldsymbol{=}\frac{\boldsymbol{K}_{\boldsymbol{B}}\boldsymbol{T}}{\boldsymbol{h}}\mathbf{exp}\left( \boldsymbol{-}\frac{\boldsymbol{\Delta G}^{\boldsymbol{\neq}}}{\boldsymbol{RT}} \right)$ (5)

$\boldsymbol{\Delta}\boldsymbol{H}^{\boldsymbol{\neq}}\mathbf{=}\boldsymbol{E}_{\boldsymbol{a}}\boldsymbol{-RT}$ (6)

$\boldsymbol{\Delta}\boldsymbol{S}^{\boldsymbol{\neq}}\boldsymbol{=}\frac{\boldsymbol{\Delta H}^{\boldsymbol{\neq}}\boldsymbol{-}\boldsymbol{\Delta G}^{\boldsymbol{\neq}}}{\boldsymbol{T}}$ (7)

Where β_i_ is the heating rate in °C/min; T_p_ is the peak temperature in the DSC trace at β_i_ in K; R is the universal gas constant (8.318 J_˙_K^-1^_˙_mol^-1^); A is the pre-exponential factor in s^-1^; E_a_ is the apparent activation energy in kJ_˙_mol^-1^; **k is the rate constant in s^-1^;** T_p0_ is the peak temperature when β_i_ is zero in K; b, c, and d is constant; T_b_ is the critical explosion temperature in K; K_B_ is Boltzmann constants (1.381×10^-23^ J_˙_K^-1^); h is Plank constant (6.626×10^-34^ J_˙_s); ΔG^≠^ is free energy of activation in kJ_˙_mol^-1^; ΔH^≠^ is enthalpy of activation in kJ_˙_mol^-1^; ΔS^≠^ is entropy of activation in J_˙_mol^-1^; T is T_p0_ calculated by Eq(2).

**References**

1 S.M. Paek, D.J. Yoo and I. Honma, Nano Lett., 2009, 9, 72.

2 A.A. Sherbini, M. Bakr, I. Ghoneim and M. Saad, J. Adv. Res., 2017, 8, 209.

3 I.Y. Jeon, Y.R. Shin, G.J. Sohn, H.J. Choi, S.Y. Bae and J. Mahmood, PNAS, 2012, 109, 5588.
